# Alternagin-C binding to α_2_β_1_ integrin controls matrix metalloprotease-9 and matrix metalloprotease-2 in breast tumor cells and endothelial cells

**DOI:** 10.1186/s40409-018-0150-2

**Published:** 2018-04-25

**Authors:** Milene Nóbrega de Oliveira Moritz, Lívia Mara Santos Eustáquio, Kelli Cristina Micocci, Ana Carolina Caetano Nunes, Patty Karina dos Santos, Tamires de Castro Vieira, Heloísa Sobreiro Selistre-de-Araujo

**Affiliations:** Department of Physiological Sciences, Federal University of São Carlos (UFSCar), São Carlos, SP 13565-905 Brazil

**Keywords:** ALT-C, α_2_β_1_ integrin, Cancer, Tumor microenvironment, MMP, *C-Myc*

## Abstract

**Background:**

Matrix metalloproteinases (MMPs) are key players in tumor progression, helping tumor cells to modify their microenvironment, which allows cell migration to secondary sites. The role of integrins, adhesion receptors that connect cells to the extracellular matrix, in MMP expression and activity has been previously suggested. However, the mechanisms by which integrins control MMP expression are not completely understood. Particularly, the role of α2β1 integrin, one of the major collagen I receptors, in MMP activity and expression has not been studied. Alternagin-C (ALT-C), a glutamate-cysteine-aspartate-disintegrin from *Bothrops alternatus* venom, has high affinity for an α2β1 integrin. Herein, we used ALT-C as a α2β1 integrin ligand to study the effect of ALT-C on MMP-9 and MMP-2 expression as well as on tumor cells, fibroblats and endothelial cell migration.

**Methods:**

ALT-C was purified by two steps of gel filtration followed by anion exchange chromatography. The α_2_β_1_ integrin binding properties of ALT-C, its dissociation constant (*K*_*d*_) relative to this integrin and to collagen I (Col I) were determined by surface plasmon resonance. The effects of ALT-C (10, 40, 100 and 1000 nM) in migration assays were studied using three human cell lines: human fibroblasts, breast tumor cell line MDA-MB-231, and microvascular endothelial cells HMEC-1, considering cells found in the tumor microenvironment. ALT-C effects on MMP-9 and MMP-2 expression and activity were analyzed by quantitative PCR and gelatin zymography, respectively. Focal adhesion kinase activation was determined by western blotting.

**Results:**

Our data demonstrate that ALT-C, after binding to α_2_β_1_ integrin, acts by two distinct mechanisms against tumor progression, depending on the cell type: in tumor cells, ALT-C decreases MMP-9 and MMP-2 contents and activity, but increases focal adhesion kinase phosphorylation and transmigration; and in endothelial cells, ALT-C inhibits MMP-2, which is necessary for tumor angiogenesis. ALT-C also upregulates *c-Myc* mRNA level, which is related to tumor suppression.

**Conclusion:**

These results demonstrate that α_2_β_1_ integrin controls MMP expression and reveal this integrin as a target for the development of antiangiogenic and antimetastatic therapies.

**Electronic supplementary material:**

The online version of this article (10.1186/s40409-018-0150-2) contains supplementary material, which is available to authorized users.

## Background

Metastasis is the main cause of death of patients with breast cancer; therefore, a full comprehension of the cell motility process is crucial for understanding how tumor dissemination occurs throughout the body [1]. The metastatic process involves several steps such as extracellular matrix (ECM) degradation, invasion, transendothelial cell migration, survival in circulation, extravasation, and colonization in a new site [2, 3]. The tumor stroma plays a fundamental role in tumorigenesis. It consists mainly of fibroblasts, ECM, vasculature, inflammatory cells and mesenchymal stem cells that, in concert with tumor cells, are responsible for secreting growth factors, proteases and chemokines to induce a continuous remodeling of the tumor microenvironment [4–6].

The ECM components play key roles in intracellular signaling by interacting with adhesion receptors such as integrins [7]. Integrins comprise a family of cell adhesion heterodimeric transmembrane receptors and their expression may vary greatly according to the environment [8–11]. α_2_β_1_ integrin is one of the major receptors for type I collagen (Col I) and it has been demonstrated to be relevant during the process of bone metastasis of prostate and breast cancer [12, 13]. The role of α_2_β_1_ integrin in the tumor microenvironment has not been fully elucidated, but the expression of β_1_ integrin subunit is altered in 30–50% of breast tumors. The β_1_ integrin subunit was shown to be required for cell proliferation, survival and invasiveness of transformed cells [14–16]. However, in vitro experiments performed on primary epithelial tumor cells have shown that the deletion of α_2_β_1_ integrin increased tumor cell intravasation and anchorage-independent growth [17].

Matrix metalloproteinases (MMPs) modify the microenvironment during tumor progression by inducing extracellular matrix remodeling and the release of cytokines and growth factors [18]. Overexpression of MMP-2 and MMP-9 is observed in various types of cancer, such as colorectal tumors, melanoma and breast cancer [19–21]. Moreover, MMP-2 and MMP-9 have been associated with tumor progression and decreased survival [22]. It has been recently demonstrated that active MMP-2 regulates vascular endothelial growth factor A (VEGF-A) expression in melanoma cells on a transcriptional level via an α_v_β_5_ integrin/phosphoinositide-3-kinase–(PI3K) dependent pathway [20], which results in activation of the endothelium, an essential step for the adhesion of circulating tumor cells. Therefore, integrin inhibition shows up as an interesting alternative for metastasis prevention.

Disintegrins are integrin inhibitors found in snake venoms [23]. Most disintegrins have the arginine-glycine-aspartate (RGD) motif, which is a very well-known ligand of α_v_β_3_ and α_5_β_1_ integrins. However, snake venoms also have another class of integrin binding proteins in which a glutamate-cysteine-aspartate (ECD) sequence replaces the RGD motif. This special class of proteins binds to α_2_β_1_ integrins and competitively inhibits cell binding to Col I [24]. Alternagin-C (ALT-C), an ECD-disintegrin-like protein, has been shown to be a potent inhibitor of collagen-induced adhesion through α_2_β_1_ integrin inhibition [24–26]. However, the effects of ALT-C on MMPs in tumor and normal cells have not been determined. Here, we provide further evidence that ALT-C binding to α_2_β_1_ integrin decreases MMP-9 and MMP-2 content in human breast cancer cells and decreases MMP-2 content in human microvascular endothelial cells (HMEC-1) by zymography. The decrease in *MMP-9* mRNA level was also confirmed by polymerase chain reaction (PCR) analysis. ALT-C also induces focal adhesion kinase (FAK) phosphorylation and upregulates *c-Myc* mRNA levels in MDA-MB-231 tumor cells. Fibroblasts were insensitive to ALT-C. These results provide new information on the roles of α_2_β_1_ integrin binding in the tumor cell and in its microenvironment.

## Methods

### Purification of alternagin-C

ALT-C was purified from *Bothrops alternatus* venom (donated by the Butantan Institute, São Paulo, Brazil) by two steps of gel filtration followed by anion exchange chromatography as previously described [24]. The purity of the final preparation was confirmed by mass spectrometry and it showed no residual proteolytic activity.

### Surface plasmon resonance (SPR)

To better characterize the α_2_β_1_ integrin binding properties of ALT-C, its dissociation constants (*K*_*d*_) relative to this integrin and to collagen I (Col I) was determined by surface plasmon resonance (SPR). The α_2_β_1_ integrin (R&D Systems) diluted in acetate buffer (20 μg/mL), pH 4.0, was immobilized to the dextran matrix of a CM5 sensor chip™ (GE Healthcare Life Sciences, Sweden) at a flow rate of 15 μL/min. This procedure resulted in ~ 1600 resonance units (RU). Collagen type I (BD Biosciences, USA) diluted in acetate buffer (30 μg/mL), pH 4.5, was similarly immobilized to the dextran matrix of a CM5 sensor chip™ at a flow rate of 15 μL/min and this procedure resulted in ~ 4000 RU.

Surfaces were activated and blocked using *N*-ethyl-*N*′-(dimethylaminopropyl) carbodiimid plus *N*-hydroxysuccinimid and ethanolamine chemistry. The chip was regenerated with Gly-HCl 2 M, pH 2.0, for 10 s. ALT-C was immediately diluted in phosphate-buffered saline (PBS – 0.05-10 μM) and injected consecutively at flow rates of 15 μL/min at 25 °C using PBS as flow buffer. Measurements were performed using equipment and supplies from BIAcore T200 (GE Healthcare Life Sciences, Sweden) and the BIA evaluation software. Kinetic parameters were analyzed using the 1:1 binding model by GraFit 7 software (Erithacus Software, England).

### Cell lines and culture

Human fibroblasts were purchased from the Cell Bank of Rio de Janeiro (Brazil) and the human breast tumor cell line MDA-MB-231 from American Type Culture Collection (ATCC, Manassas, USA). Both cell lines were maintained in Dulbecco’s Modified Eagle Medium (DMEM – Vitrocell, Brazil) supplemented with 10% (*v*/*v*) fetal bovine serum (FBS). Human dermal (foreskins) microvascular endothelial cells HMEC-1 from (ATCC CRL-3243) were cultured in MCDB-131 (Sigma, Brazil). All cell lines were cultured in the presence of penicillin (100 IU/mL), streptomycin (100 μg/mL) and l-glutamine (2 mM) in a humidified environment with 5% CO_2_ at 37 °C. For the cell passages, 0.25% trypsin (Sigma-Aldrich, USA), 0.1% ethylenediamine tetraacetic acid (EDTA – Sigma-Aldrich, USA) solution was used.

### Flow cytometry analysis

The profile of α2 and β1 integrin subunits of each cell line was determined by flow cytometry using specific monoclonal antibodies: anti-α_2_ (LSBio-C188740, USA), and anti-β_1_ (SC-13590, Santa Cruz Biotechnology). Briefly, 1 × 10^6^ cells were incubated with 1 μg of antibodies at 4 °C for 30 min. Then, cells were washed with PBS and centrifuged at 4 °C for 10 min at 150×*g*. Next, 0.5 μg of fluorescein isothiocyanate anti-IgG (SC-2010, Santa Cruz Bioechnology) was added to each sample and incubated for 30 min at 4 °C in absence of light. After, cells were washed again with PBS, centrifuged and immediately analyzed in a FACSCalibur flow cytometer (BD Bioscience, USA).

### Transendothelial migration assay

This assay mimics tumor cell migration through endothelial blood cells, one of the crucial steps in metastasis. HMEC-1 cells were seeded (1 × 10^5^) in 8-μm pore inserts (12 wells/plate) (BD Biosciences, USA) and cultured in medium containing 10% FBS until they achieved confluence (48 h) and formed monolayers. During this period, the wells under inserts contained medium without FBS. MDA-MB-231 cells were stained with PKH26 red fluorescent cell linker (Sigma-Aldrich, USA), treated or not with ALT-C and placed in the upper chamber covered with a monolayer of HMEC-1.

The stained tumor cells were placed in the inserts with medium without FBS and under the inserts was added medium containing 5% FBS (chemoattractant, Vitrocell, Brazil). After incubation for 16 h at 37 °C, 5% CO_2_, the transmigrated cells were fixed with 3.7% formaldehyde and stained with DAPI. Ten randomly chosen fields in the inserts were photographed and cells counted using a fluorescence microscope (Olympus U-RFL-T, 20× objective, DP2BSW software, Japan).

### Wound healing migration assay

A wound healing migration assay measures the repopulation of wounded cultures. Cells were seeded into 12-well culture plates at 1 × 10^5^ cells/well and cultured in medium containing 10% FBS to achieve monolayer confluence. The monolayers were carefully wounded using a 200-μL pipette tip, and cellular debris was removed by washing with medium. The wounded monolayers were then incubated for 24 h in serum-free medium (SFM) containing 0–1000 nM of ALT-C. Images immediately after wounds (*t* = 0 h) were captured to record the initial area, and the recovery of the wounded monolayers due to cell migration toward the denuded area was evaluated at 24 h (*t* = Δ h).

The images were captured using an inverted microscope (Olympus CK2 ULWCD 0.30; 10× objective, Japan) equipped with a digital camera (Cool SNAP-Pro Color with Image Pro software). The area of the wound was quantified using Java’s Image J software (http://rsb.info.nih.gov) in the polygon selection mode. The migration of cells toward the wound was expressed as a percentage of wound closure: percent of wound closure = [(At = 0 h – At = Δ h)/At = 0 h] × 100%, where At = 0 h is the area of the wound measured immediately after scratching, and At = Δ h is the area of the wound measured 24 h after scratching.

### Gelatin zymography assay

The content of MMPs on conditioned media from the wound healing assay was analyzed by gelatin zymography as previously described [27] with some modifications. After treatment with ALT-C, the culture medium was collected, centrifuged at 10,000×*g* for 5 min at 4 °C and incubated in sample buffer under non-reducing conditions. The samples were maintained in ice and immediately loaded (20 μg) in the gels. The samples were resolved on a 10% polyacrylamide gel containing 0.1% gelatin at 4 °C. The gel was washed two times with 2.5% Triton Χ-100 and incubated at 37 °C for 18 h in 50 mM Tris buffer, pH 8.0, 5 mM CaCl_2_, 0.02% NaN_3_ and 10 mM ZnCl_2_. After staining with Coomassie Blue R-250 and distaining with acetic acid:methanol:water (1:4:5), the clear bands were quantified by densitometry using Image J software. MMP-2 and MMP-9 were quantified in arbitrary units (AU) using GraphPad Prism 5.0 software (La Jolla, USA).

### Isolation of total RNA and synthesis of cDNA

Cells were seeded in 6-cm dishes (Corning, USA) in culture medium (DMEM or MCDB-131, Brazil) plus 10% FBS for 48 h at 37 °C and 5% CO_2_. The cells were then incubated with 10, 100 or 1000 nM ALT-C. After 24 h, culture medium was removed and cells were lysed with cold TRIzol Reagent (Invitrogen, USA) according to the manufacturer’s protocol for total RNA isolation. RNA concentrations and purity were determined by the ratio of the absorbance at 260 and 280 nm using a Nanodrop 2000 the RNA integrity was confirmed on 1% agarose-formaldehyde gel stained with ethidium bromide.

Total RNA was reverse transcribed into cDNA using M-MLV Reverse Transcriptase (Promega, USA). cDNA was stored at − 20 °C until use. Oligonucleotide primers were designed using Primer Blast (http://www.ncbi.nlm.nih.gov/tools/primer-blast/). The primer sequences were: *GAPDH* forward 5′ GATGCTGGTGCTGAGTATGT and reverse 5′ GTGGTGCAGGATGCATTGCT; *c-Myc* forward 5′ CCTACCCTCTCAACGACAGC and reverse 5′ CTTGTTCCTCCTCAGAGTCGC; *MMP-2* forward 5’ AGGACCGGTTCATTTGGCGG and reverse 5′ TGGCTTGGGGTACCCTCGCT; *MMP-9* forward 5’ CGCTACCACCTCGAACTTTG and reverse 5′ GCCATTCACGTCGTCCTTAT.

### Analysis by quantitative polymerase chain reaction (qPCR)

The amplification mixtures contained 6.25 μL of the fluorescent dye Eva Green Supermix (Bio-RadUSA), 0.5 μL of cDNA, 4.75 μL of diethyl dicarbonate (DEPC) water and 1 μL (100 μM/μL) of each primer in a final volume of 12.5 μL. Thermal cycling conditions for all genes were 15 min at 95 °C followed by 45 cycles of 30 s at 72 °C and 30 s at 56 °C for *GAPDH*, 59.5 °C for *c-Myc*, 60 °C for *MMP-2* and 59 °C for *MMP-9*, respectively. For each gene, all samples were amplified simultaneously in duplicate in one assay run. Data were analyzed using the comparative cycle threshold (*C*t) method. The target RNA level was normalized to *GAPDH* RNA level as previously described [28]. A blank sample containing water, primers and Eva Green but no template was also included.

### Western blotting analysis

MDA-MB-231 cells were seeded (10^5^ cells/well) in a six-well plate in culture medium (DMEM) plus 10% FBS overnight at 37 °C and 5% CO_2_ and then incubated with 10, 100 or 1000 nM ALT-C. After 24 h, culture medium was removed and cells were lysed with RIPA buffer [150 mM NaCl; 50 mM Tris; pH 8.0; 0.1% sodium dodecyl sulfate (SDS); 1% Triton Χ-100] and proteases and phosphatases inhibitors. Protein quantitation was carried out using the BCA Protein Assay kit (Thermo Scientific, USA), according to the supplier’s instructions.

Thirty micrograms of each sample were diluted in denaturing sample buffer containing glycerol, SDS, dithiothreitol (DTT) and bromophenol blue. After electrophoresis, the samples were transferred to nitrocellulose membranes and blocked with skimmed milk powder (4%). Rabbit monoclonal antibodies against p-FAK (ab81298) and FAK (ab40794) were used at 1:1000 dilution in PBS. A secondary anti-rabbit antibody (ab97051) was used at 1:10,000 dilution in milk powder. Detection of proteins was performed using the Chemiluminescent Peroxidase Substrate-1 (SLBJ1875, Sigma-Aldrich, USA). Images were obtained on a digital documentation system (Chemi-Doc Xr, Bio-Rad Lab, USA) and relative quantitation was done by densitometric analysis of the images using the Image J software and normalizing to GAPDH band densities when indicated.

### Statistical analysis

Each experiment was repeated twice (*n* = 2) in triplicate and the mean and standard errors were calculated. The results were compared statistically using one-way analysis of variance (ANOVA) and Tukey’s test was used when *p* values were * p < 0.05, ** p < 0.01 or *** p < 0.001*.* Statistical comparisons were done in relation to the 0 nM condition.

## Results

### ALT-C affinity to α_2_β_1_ integrin was higher than to collagen I

**Fig. 1:**
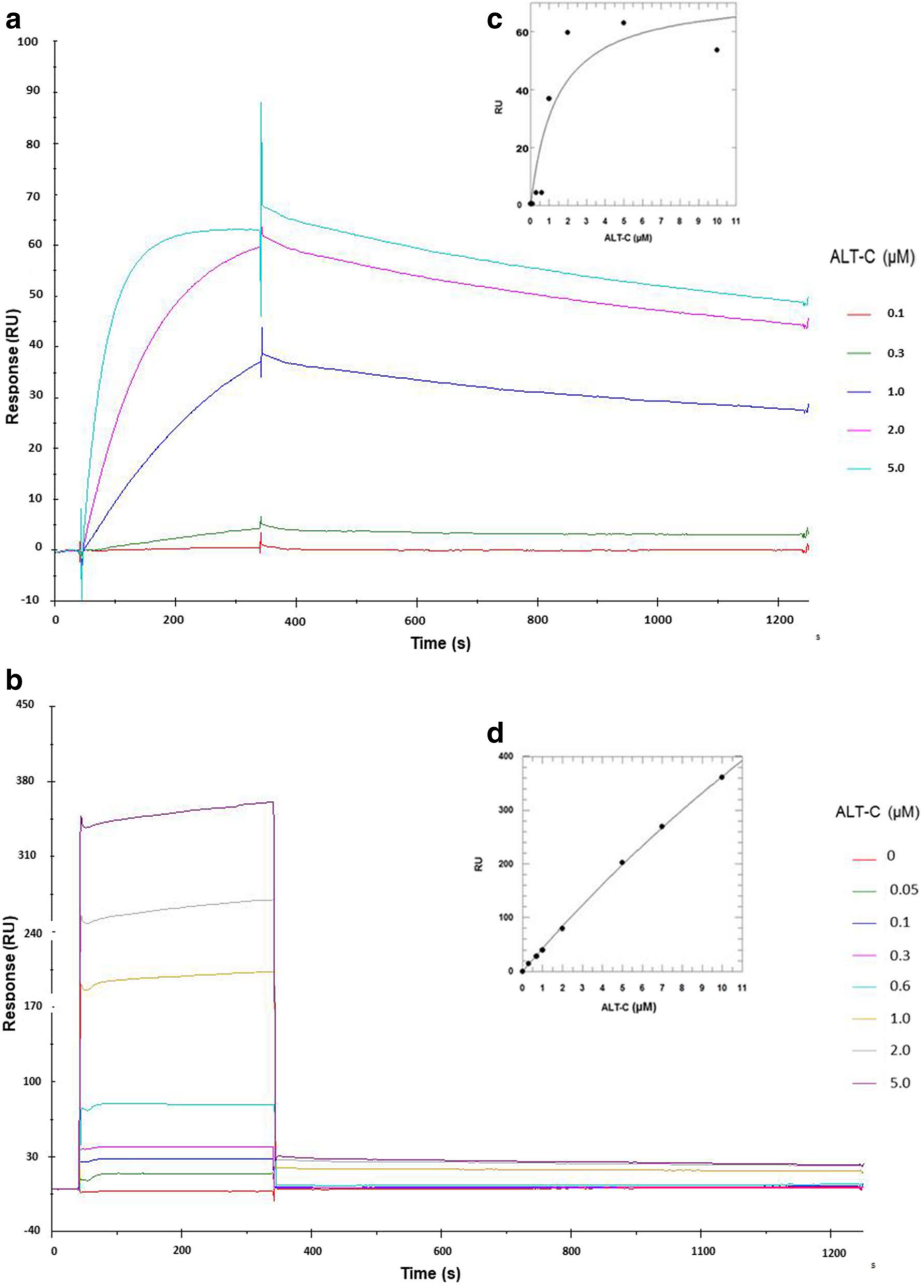
Characteristics of ALT-C binding to α_2_β_1_ integrin or collagen type I (Col I). Representative sensorgrams and dose-dependent binding of ALT-C (0.0–5.0 μM) measured by surface plasmon resonance (SPR): (**a**) to α_2_β_1_ integrin; and (**b**) to Col I. Kinetic curves were analyzed using the 1:1 binding model by GraFit 7 software for: (**c**) α_2_β_1_ integrin; and (**d**) Col I

Our group has previously demonstrated the binding of ALT-C to α_2_β_1_ integrin by inhibition of cell adhesion assays in Col I coating; however, the kinetics parameters of this association were never determined. In addition, it has been suggested that ECD-proteins bind directly to collagen as well as to α_2_β_1_ integrin, which could have implications in their mechanism of action. To address the question if ALT-C could bind to Col I and to α_2_β_1_ integrin, we determined the ALT-C dissociation constant (*K*_*d*_) values for α_2_β_1_ integrin and Col I by surface plasmon resonance (SPR). α_2_β_1_ integrin and Col I were immobilized to a carboxymethylated dextran (CM5) sensor chip™ and ALT-C was passed over the layers in PBS buffer. ALT-C bound to α_2_β_1_ integrin with a *K*_*d*_ *≈* 1.4 μM (Fig. 1a); in contrast, a lower affinity was found for Col I, with a *K*_*d*_ *≈* 48 μM (Fig. 1b). ALT-C binding to α_2_β_1_ integrin was approximately 35 times stronger than to Col I.

The SPR was also performed for ALT-C binding to α_v_β_3_, α_5_β_1_ and fibronectin (FN). As expected, ALT-C did not bind to α_v_β_3_ and α_5_β_1_ integrins or to FN, confirming the specificity of ALT-C for α_2_β_1_ and Col I (Additional file 1).

### Characterization of integrin cell expression by flow cytometry

The expression of the α_2_β_1_ integrin subunits on cell surfaces was analyzed by flow cytometry. The three cell lines used in this work have similar expression profiles of α_2_β_1_ integrin with high contents of α_2_ and β_1_ integrin subunits (Additional file 2). Therefore, these cells were considered comparable models to investigate the role of α_2_β_1_ integrin on MMPs and cell migration. ALT-C treatment (10–1000 nM) did not change the α_2_ subunit content of human breast adenocarcinoma cells (MDA-MB-231), which was also confirmed by Western blotting (Additional file 3).

### ALT-C increased MDA-MB-231 cell trans-endothelial migration

**Fig. 2:**
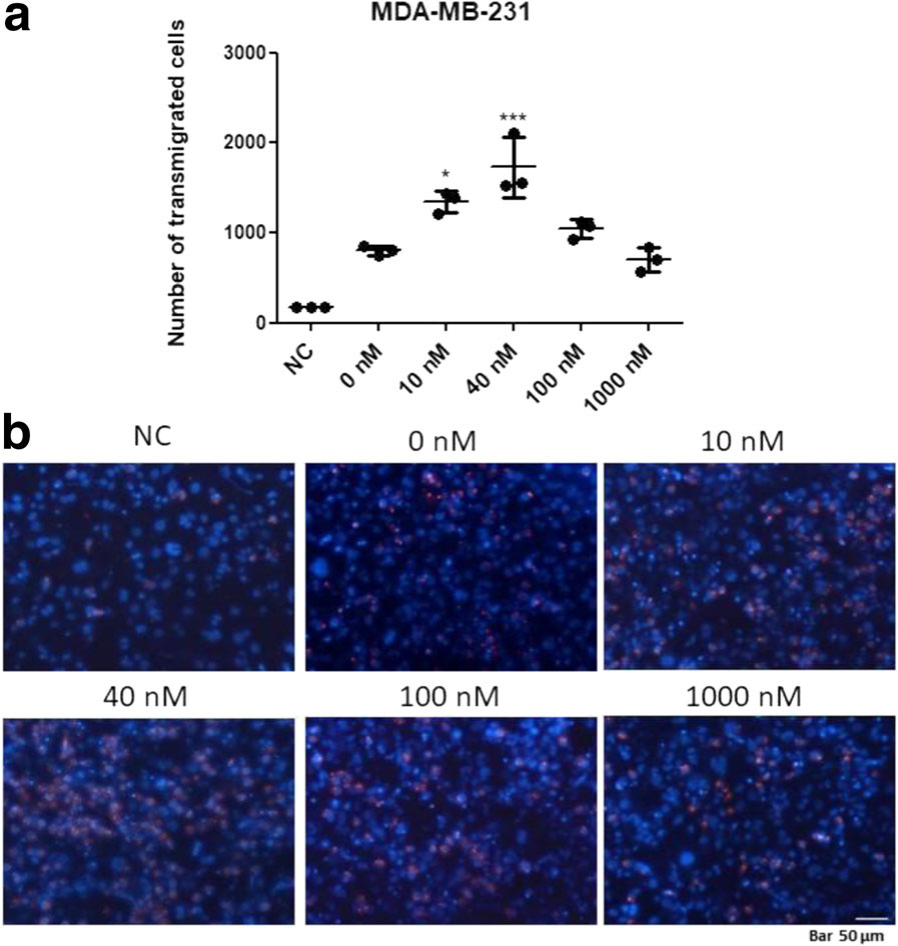
ALT-C stimulates the transmigration of MDA-MB-231 cells through a monolayer of HMEC-1 cells. **a** MDA-MB-231 cells were plated in wells containing HMEC-1 cells, and 5% of fetal bovine serum (FBS) was used as a chemoattractant in the lower chamber. Tumor cells were first stained with PKH26 Red Fluorescent Cell Linker and after 16 h of transmigration assay the cells were fixed, stained with DAPI and counted (an average of eight fields from each treatment). Negative control means the assay in the absence of FBS in the lower chamber. The assay was performed in triplicate with two independent assays (*n* = 2). The results were compared using ANOVA followed by Tukey’s test (* *p* < 0.05, and *** *p* < 0.001). **b** Representative images of transmigrated cells of each treatment. Bar represents 50 μm

Since disintegrins and disintegrin-like proteins are known to competitively inhibit cell migration, we first investigated whether α_2_β_1_ integrin could be a relevant player for tumor cell transmigration using ALT-C as a α_2_β_1_ integrin ligand. ALT-C increased tumor cell transmigration through a layer of endothelial cells at concentrations of 10 and 40 nM by 67.9% and 116.5%, respectively (Fig. 2a). From a concentration of 100 nM and higher, ALT-C lost this ability. Tumor cells were labeled using the PKH26 Red Fluorescent Cell Linker kit for General Cell Membrane Labeling^®^ (Sigma-Aldrich, USA) to distinguish them from HMEC-1 cells. All cell nuclei were stained with 4′,6-diamidino-2-phenylindole (DAPI) (Fig. 2b).

### ALT-C inhibited MMPs in the conditioned media of cell cultures from a wound healing assay

**Fig. 3:**
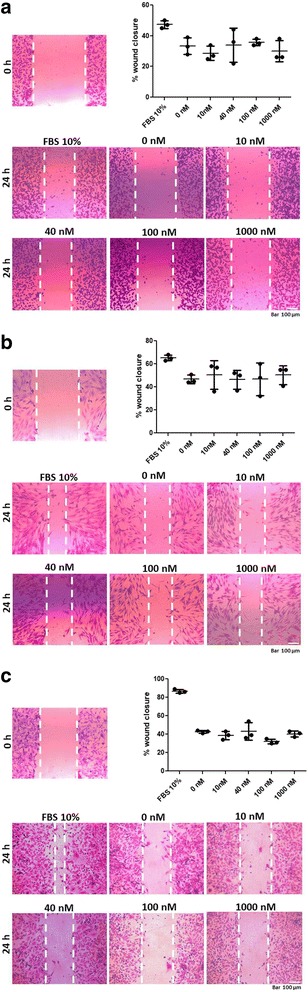
ALT-C effects on: (**a**) MDA-MB-231 cells; (**b**) fibroblasts; and (**c**) HMEC-1 cells were plotted as a percentage of wound closure 24 h after wounding. FBS 10% represents cells in presence of medium with FBS (10%) as positive control. The assay was performed in triplicate with two independent assays (*n* = 2). *p* values were determined using ANOVA followed by Tukey’s test, considering significant when *p* < 0.05. Representative photos of wounds were taken at time zero and 24 h after wounding. Cells were stained with crystal violet 0.1%

To study the role of α_2_β_1_ integrin in MMP content, we tested ALT-C in a wound healing assay, which is another commonly used migration assay. No ALT-C effect was observed on the migration ability of MDA-MB-231 cells (Fig. 3a), fibroblasts (Fig. 3b) or HMEC-1 cells (Fig. 3c) in wound healing assays. These results suggest that the ALT-C effects may be depend on one or more FBS components to stimulate migration, since ALT-C acts on cell migration through endothelial cells in presence of FBS (Fig. 2).

**Fig. 4:**
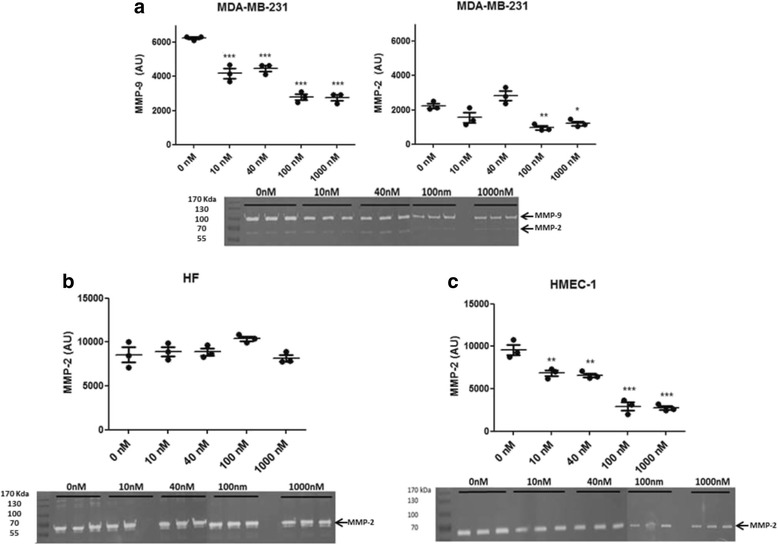
Effects of ALT-C on the MMP-9 and MMP-2 contents in the conditioned media from (**a**) MDA-MB-231, (**b**) human fibroblasts, and (**c**) HMEC-1 cells. MMP content was detected by band densitometry in the conditioned media (peak area) after wounding and incubation with ALT-C for 24 h. MMP-2 and MMP-9 contents were determined by band densitometry. The assay was performed in triplicate with two independent assays (*n* = 2). * *p* < 0.05, ** *p* < 0.01, *** *p* < 0.001 compared to control (without ALT-C)

The total content of MMP-9 and MMP-2 in the conditioned media from ALT-C-treated cells was analyzed by gelatin zymography after the wound healing assay. ALT-C significantly decreased MMP-9 content in the conditioned media from MDA-MB-231 cell culture at all concentrations used (Fig. 4a). MMP-2 content was also decreased in MDA-MB-231 cells but only at the 100 and 1000 nM concentrations of ALT-C. MMP content was not changed in the conditioned media from human fibroblast culture treated with ALT-C (Fig. 4b). All concentrations of ALT-C (10, 40, 100 and 1000 nM) significantly decreased MMP-2 level in HMEC-1 cells (Fig. 4c). MMP-9 was not detected in the conditioned media of fibroblasts or HMEC-1 cells.

### ALT-C inhibited MMP mRNA level in tumor cells

**Fig. 5:**
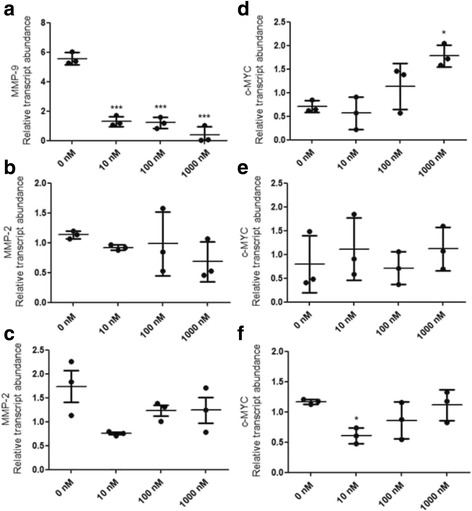
(**a**) ALT-C effects on *MMP-9* expression in MDA-MB-231 cells, and (**b**) on *MMP-2* mRNA levels in fibroblasts or (**c**) HMEC-1 cells. Levels of *c-Myc* mRNA after ALT-C treatment in (**d**) MDA-MB-231 cells, (**e**) human fibroblasts, and (**f**) HMEC-1 cells. The values represent relative transcript abundance and the *p* value was determined using ANOVA followed by Tukey’s test. Values were normalized to the level of glyceraldehyde 3-phosphate dehydrogenase (GAPDH) mRNA. The assay was performed in triplicate with two independent assays (*n* = 2). * *p* < 0.05, *** *p* < 0.001 compared to control (0 nM)

To further investigate the effects of ALT-C on *MMP* levels, we determined the *MMP-2* and *MMP-9* mRNA levels by quantitative PCR. *MMP-9* level in MDA-MB-231 cells was strongly inhibited by ALT-C at all tested concentrations (10, 100 and 1000 nM; Fig. 5a), which corroborated the zymography results from the conditioned media. Accordingly, ALT-C did not affect *MMP-2* level in human fibroblasts (Fig. 5b). However, *MMP-2* level was not changed in endothelial cells (Fig. 5c), in contrast with the zymography results.

### ALT-C induced the increase of c-Myc mRNA level in MDA-MB-231 cells

The oncogene *c-Myc* has been associated with the expression of integrin genes in cells from different tissues and its overexpression significantly inhibited the migration and invasiveness of MDA-MB-231 cells in vitro [29]. Therefore, we investigated whether ALT-C could trigger the increase of *c-Myc* mRNA levels upon α_2_β_1_ integrin binding. ALT-C upregulated *c-Myc* mRNA level at 1000 nM in MDA-MB-231 cells (Fig. 5d). However, no effect on *c-Myc* mRNA level was observed in human fibroblasts (Fig. 5e). Conversely, ALT-C inhibited *c-Myc* mRNA level in HMEC-1 cells at 10 nM (Fig. 5f).

### A low concentration of ALT-C induced FAK phosphorylation in MDA-MB-231 cells

**Fig. 6:**
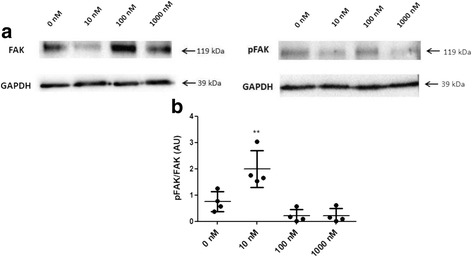
ALT-C induces FAK phosphorylation at 10 nM but not at 100 and 1000 nM. **a** FAK and p-FAK protein level revealed by Western blotting in lysates extracted from MDA-MB-231 treated with ALT-C (10, 100 and 1000 nM) and control (0 nM). **b** Values represent the normalized densitometry ratio of p-FAK and FAK and the *p* value was determined using ANOVA followed by Tukey’s test. Values were also previously normalized to the level of GAPDH densitometry. ** *p* < 0.01 compared to control (0 nM). The assay was performed in independent assays (*n* = 2) and Western blotting in quadruplicate

To address whether ALT-C was activating α_2_β_1_ integrin we sought to determine the content of phosphorylated FAK (p-FAK), which is a hallmark of integrin activation. After 24 h of incubation, ALT-C resulted in highly phosphorylated FAK at 10 nM, but not at 100 or 1000 nM in MDA-MB-231 cells (Fig. 6).

## Discussion

Previous studies from our group have shown that ALT-C specifically inhibits the adhesion of α_2_β_1_-overexpressing human chronic myelogenous leukemia cells (K562) to collagen I with a half-maximal inhibitory concentration (IC_50_) of 100 nM [24]. Later, it was reported that jararhagin-C, an ALT-C homolog from *Bothrops jararaca* venom, also binds directly to collagen through its disintegrin domain [30]. However, the relevance of this collagen-binding property to the mechanism of action of such proteins was not well understood. To further elucidate this mechanism, we determined the *K*_*d*_ of ALT-C to both collagen and its receptor by plasmon resonance. Our data corroborate those of Souza et al. [24], confirming the strong interaction between ALT-C and α_2_β_1_ integrin (*K*_*d*_ ≈ 1.4 μM). ALT-C affinity to collagen was much lower (*K*_*d*_ ≈ 48 μM). Therefore, it was demonstrated that the predominant target of ALT-C is α_2_β_1_ integrin. Using the same approach, we have recently reported that a disintegrin from *Bothrops alternatus* (Dis*Ba*-01), a recombinant RGD-disintegrin from *Bothrops alternatus*, binds to α_V_β_3_ and α_5_β_1_ integrins with high affinity (*K*_*d*_ = 4.63 × 10^− 7^ and 7.62 × 10^− 5^ M, respectively) [31]. Therefore, the affinity of α_2_β_1_ integrin for ALT-C is approximately five times higher than of α_5_β_1_ integrin for an RGD disintegrin.

ALT-C in low concentrations increased the transmigration of MDA-MB-231 cells through an endothelial cell layer. This assay is intended to simulate the extravasation of tumor cells through blood vessels that leads the establishment of metastasis [17]. Previous results demonstrated a potent chemotactic effect of ALT-C on neutrophils, an effect mediated by FAK and PI3K activation [32]. ALT-C also increased protein kinase B (Akt/PKB) phosphorylation in endothelial cells, which is a key signaling pathway for cell survival [25]. However, higher ALT-C concentrations did not produce the same chemotactic effect. This bell-shape result of the concentration-effect curve was previously observed in our first study with ALT-C [33], and might be due to receptor internalization. Higher concentrations of ALT-C also inhibited FAK phosphorylation, in agreement with the transmigration results. The activation of the FAK/PI3K/Akt axis results in the phosphorylation of several proteins involved in the polymerization and stabilization of the actin cytoskeleton that are necessary for cell migration [34, 35]. Our data suggest that, at low concentrations, ALT-C binding to α_2_β_1_ integrin triggers integrin-mediated intracellular signaling events such as FAK phosphorylation and stimulation of cell transmigration. However, at higher concentrations, FAK is not activated, and the tumor cells lose the ability to transmigrate.

We did not observe any effects of ALT-C in the wound healing assay, and then we tested the MMP-2 and MMP-9 contents in the conditioned media from these experiments. We observed that ALT-C decreased the content of both enzymes in the supernatants of the MDA-MB-231 cultures, and that of MMP-2 in the HMEC cultures, without any effect on the MMP content on the fibroblast cultures. These results suggest that wound closure in this assay does not depend on MMPs. We have not analyzed MMP content in the supernatants from the transmigration assays due to the presence of serum in the media, which causes strong interference in the zymographic analysis. However, the possibility that cells are transmigrating in an MMP-independent way should be considered as previously suggested by others [36, 37].

MMPs can be regulated by integrins through different pathways related to ECM remodeling. A study showed that function-blocking anti-α_3_ antibodies decrease MMP-9 activity in MDA-MB-231 cells [38]. The α_3_β_1_ integrin seems to regulate the selection of a specific polyadenylation site within the *MMP-9* mRNA via the activation of an integrin-mediated extracellular signal-regulated kinase (MEK/ERK) signaling pathway, resulting in the generation of a short and more stable transcript and the subsequent synthesis of MMP-9 protein [39]. When the integrin is blocked or silenced, MEK/ERK signaling is decreased and a longer mRNA is produced that is more easily subjected to degradation without the generation of MMP-9 protein. We believe that a similar mechanism would also be possible for α_2_β_1_ integrin, although this hypothesis has not been confirmed.

For tumor growth, new vessels are necessary to maintain its nutrition. The process of tumor angiogenesis involves several steps of cell–cell and cell–matrix interactions that allow endothelial cells to migrate towards the tumor. Proteases are also required to cleave the basement membrane and interstitial matrix molecules, including MMPs [40]. Among the MMPs, MMP-2 has been described as playing a key role in angiogenesis and in the invasiveness phenotype as well [40–42]. We have previously demonstrated that ALT-C modulates fibroblast growth factor (FGF)-induced angiogenesis in vivo using the Matrigel plug model in nude mice [33]. In that study, we observed that low concentrations of ALT-C are pro-angiogenic, but high concentrations such as 1000 nM, completely inhibited angiogenesis. In the present study, we demonstrated that ALT-C decreased MMP-2 protein level in endothelial cells at all tested concentrations, which could be prejudicial to the formation of new vessels since MMP-2 is required for angiogenesis. Our data corroborate other studies in which MMP-2 downregulation in cancer cells and *Mmp2*-deficient mice show reduced angiogenesis and tumor growth [43]. It is possible that ALT-C is interfering with the mechanisms underlying MMP-2 activation, but additional assays are required to address this question.

Oncogenes such as *c-Myc* are involved in metastasis by affecting a number of cellular processes, including cell growth, proliferation and apoptosis [44, 45]. Paradoxically, studies have demonstrated that c-*Myc* can also act as a tumor suppressor and is related to integrins in cell adhesion and migration [29]. Recently, it was reported that α_1_β_1_ collagen receptor expression is controlled by *c-Myc* in colorectal cancer cells [46]. However, the mechanisms underlying the control of *c-Myc* RNA level are not well understood. Some studies have demonstrated that *c-Myc* transcription is related to an AKT-dependent mechanism [47, 48], while others have shown that FAK is associated with the PI3 kinase/AKT pathway in tumor progression [49, 50]. More recently, it was reported that PI3 Kinase/AKT signaling promotes *c-Myc* activation [51].

Previous results demonstrated the relation between ALT-C binding to α_2_β_1_ integrin and the activation of the PI3 kinase/AKT axis and activation of FAK as well [25, 32]. ALT-C (1000 nM) upregulated *c-Myc* mRNA level in MDA-MB-231 cells, but not in fibroblasts. Surprisingly, *c-Myc* mRNA level in endothelial cells was downregulated by 10 nM of ALT-C, but not by higher concentrations. *c-Myc* is considered an oncogene, and the overexpression of *c-Myc* significantly inhibited migration and reduced the invasiveness of MDA-MB-231 cells in vitro [29]. On the other hand, Magid et al. [52] suggested that *c-Myc* activates MMP-9 RNA level in endothelial cells under shear stress. Since *c-Myc* is related to the transcription of integrin genes in cells from different tissues [29, 53], we hypothesize that ALT-C binds to and activates α_2_β_1_ integrin upregulating *c-Myc* mRNA level via activation of the FAK/PI3K/AKT axis.

**Fig. 7:**
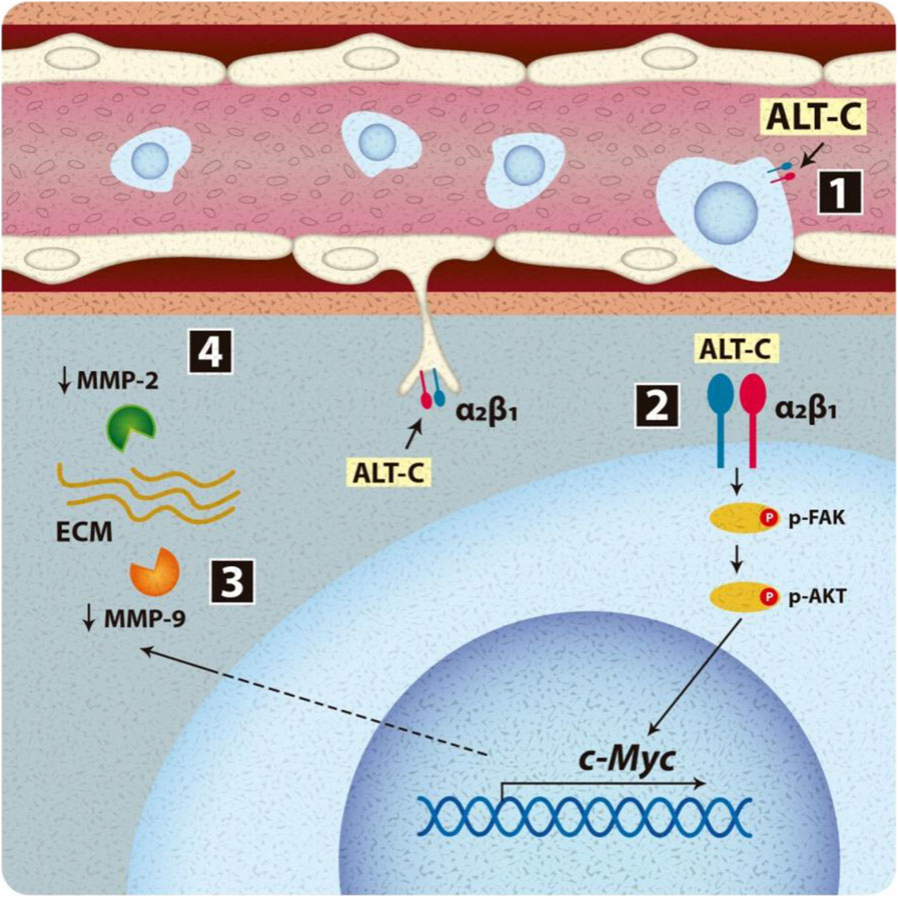
Potential mechanism of ALT-C in the tumor microenvironment and cancer progression. (**1**) Transmigration of breast tumor cells (in blue) through endothelial cells is induced by low concentrations of ALT-C. (**2**) ALT-C binds to α_2_β_1_ integrin, triggering *c-Myc* upregulation via p-FAK and p-AKT activation resulting in downregulation of the proteins (**3**) MMP-9 and (**4**) MMP-2, thus decreasing tumor cell invasion into ECM. ALT-C also acts via α_2_β_1_ integrin on endothelial cells, decreasing MMP-2, which inhibits the formation of new blood vessels

Studies have shown that the overexpression of *c-Myc* and of α_2_β_1_ integrin reduces invasion and metastasis in MDA-MB-231 breast tumor cells [17, 29]. Together, these results indicate a role for α_2_β_1_ integrin in *c-Myc* activation and tumor progression. Thus, Fig. 7 provides the potential ALT-C mechanisms. To the best of our knowledge, this is the first report of *c-Myc* upregulation by α_2_β_1_ integrin activation after ligand binding.

## Conclusions

In summary, these results suggest that ALT-C binds to α_2_β_1_ integrin in tumor cells and inhibits MMP-9 and MMP-2, but upregulates c*-Myc* (mRNA level). In endothelial cells, ALT-C decreases MMP-2 content required for angiogenesis as demonstrated by zymography. Fibroblasts are insensitive to this integrin, at least regarding the activities studied in this work. Based on the present study, we propose that ALT-C interferes with the tumor progression by binding to the α_2_β_1_ integrin tumor cells. It is also hypothesized that ALT-C impairs angiogenesis by reducing MMP-2 content in endothelial cells. All together, these results highlight the possibilities of interfering in the tumor microenvironment and consequently in tumor progression by considering α_2_β_1_ integrin as a target against metastasis.

## Additional files

Additional file 1: Characteristics of ALT-C binding to α_v_β_3_ and α_5_β_1_ integrins or fibronectin. Representative sensorgrams and dose-dependent binding of ALT-C (0.0–7.0 μM) measured by SPR to (**A**) α_v_β_3_ integrin, to (**B**) α_5_β_1_ integrin and to (**C**) fibronectin. (DOCX 109 kb)

Additional file 2: Integrin content analysis by flow cytometry on MDA-MB-231 cells, human fibroblasts and HMEC-1. (DOCX 59 kb)

Additional file 3: (**A**) Expression of α_2_ integrin subunit by western blotting in lysates extracted from MDA-MB-231 treated with ALT-C. (**B**) The values represent the normalized densitometry ratio of α_2_ and GAPDH expression. *p* value was determined using ANOVA followed by Tukey’s test. (DOCX 41 kb)

## Abbreviations

ALT-C: alternagin-C
ANOVA: one-way analysis of variance
Col I: type I collagen
DAPI: 4′,6-diamidino-2-phenylindole
DMEM: Dulbecco’s Modified Eagle Medium
DTT: dithiothreitol
ECM: extracellular matrix
EDTA: ethylenediamine tetraacetic acid
FAK: focal adhesion kinase
FBS: fetal bovine serum
FGF: fibroblast growth factor
FN: fibronectin
GAPDH: glyceraldehyde 3-phosphate dehydrogenase
MMP-2: matrix metalloproteinase 2
MMP-9: matrix metalloproteinase 9
MPP: matrix metalloproteinase
PBS: phosphate-buffered saline
PCR: polymerase chain reaction
qPCR: quantitative polymerase chain reaction
RU: resonance units
SDS: sodium dodecyl sulfate
SFM: serum-free medium
SPR: surface plasmon resonance
SPR: surface plasmon resonance
