# Supplementary figures and images for: Alternagin-C binding to α2β1 integrin controls matrix metalloprotease-9 and matrix metalloprotease-2 in breast tumor cells and endothelial cells
Source: J Venom Anim Toxins Incl Trop Dis. 2018 Apr 25;24:13. doi: 10.1186/s40409-018-0150-2 (PMC5917863; doi:10.1186/s40409-018-0150-2)

**Additional file 1**

**
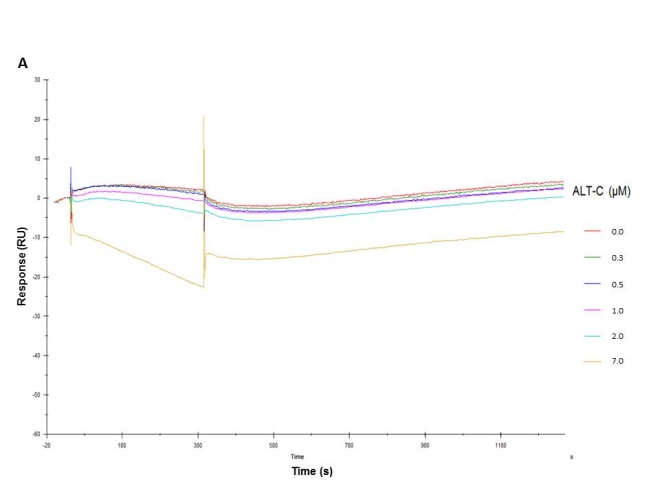
**

**
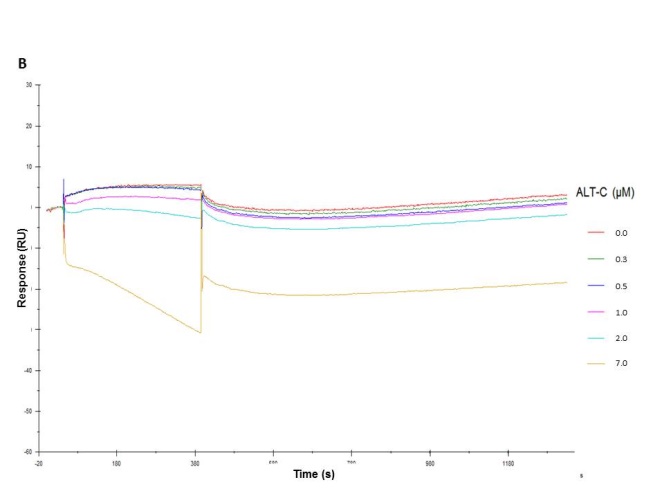
**

**
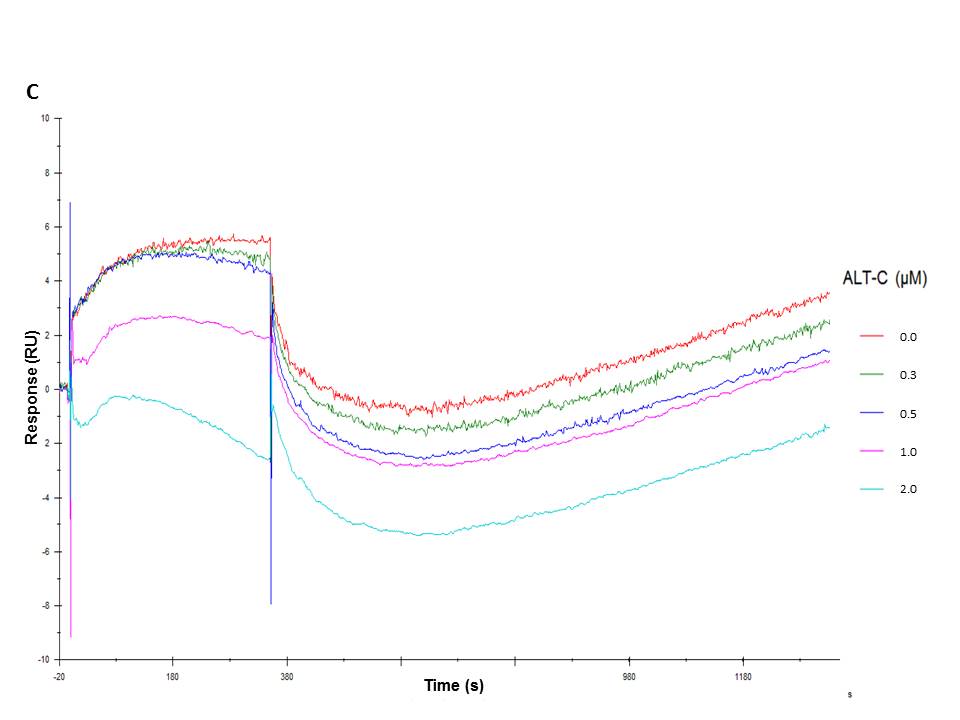
**

Supplement: Supplementary file 1 — Characteristics of ALT-C binding to αvβ3 and α5β1 integrins or fibronectin. Representative sensorgrams and dose-dependent binding of ALT-C (0.0–7.0 μM) measured by SPR to (A) αvβ3 integrin, to (B) α5β1 integrin and to (C) fibronectin. (DOCX 109 kb) [file 40409_2018_150_MOESM1_ESM.docx]

**Additional file 2**


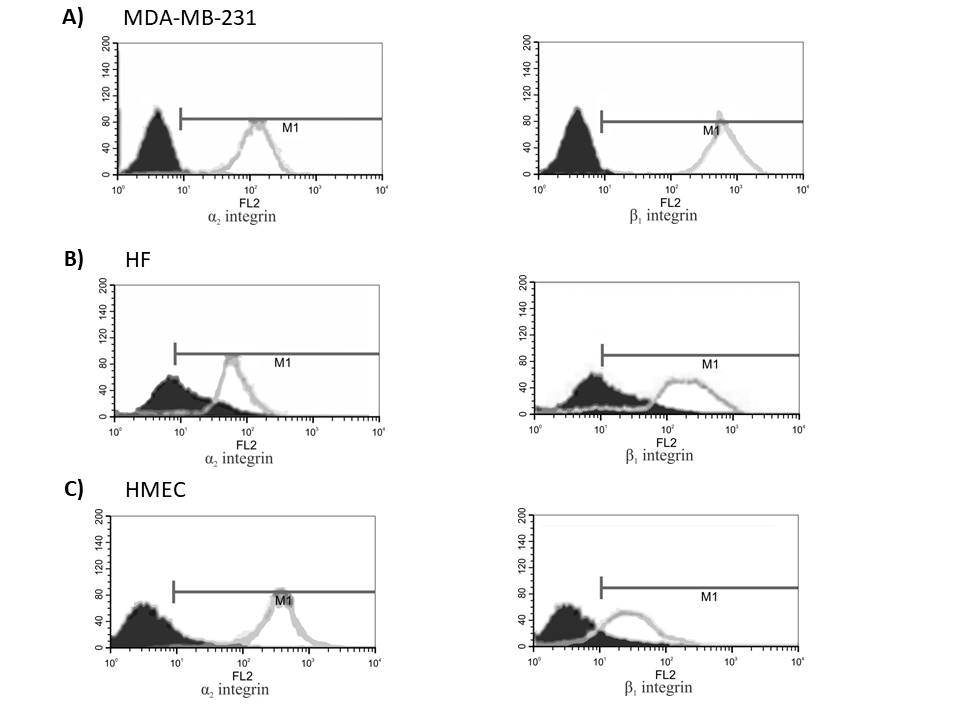

Supplement: Supplementary file 2 — Integrin content analysis by flow cytometry on MDA-MB-231 cells, human fibroblasts and HMEC-1. (DOCX 59 kb) [file 40409_2018_150_MOESM2_ESM.docx]

**Additional file 3**


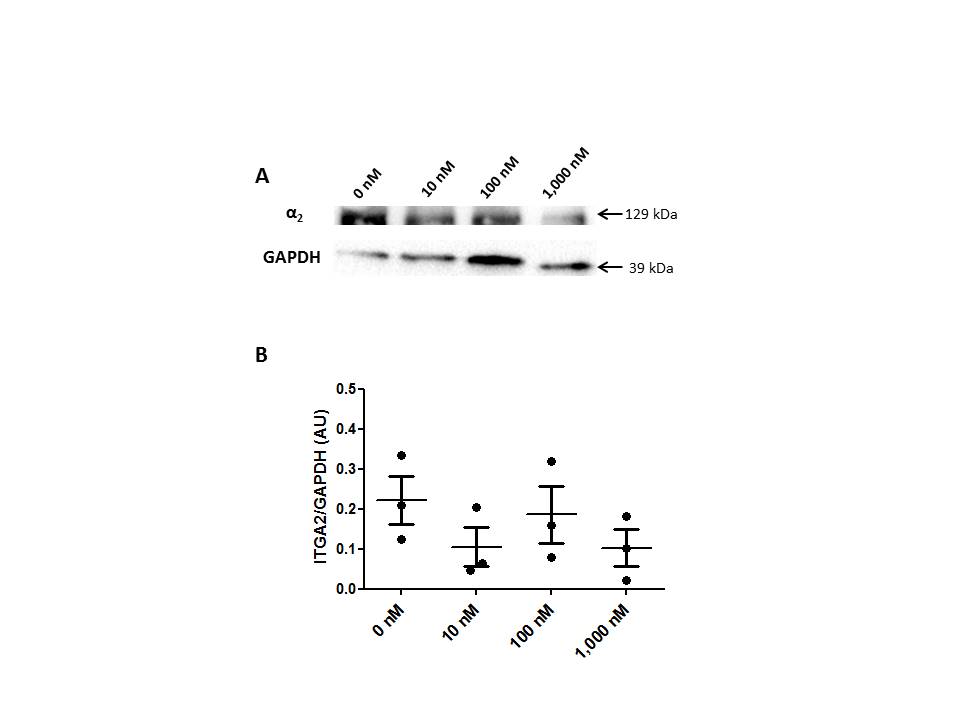

Supplement: Supplementary file 3 — (A) Expression of α2 integrin subunit by western blotting in lysates extracted from MDA-MB-231 treated with ALT-C. (B) The values represent the normalized densitometry ratio of α2 and GAPDH expression. p value was determined using ANOVA followed by Tukey’s test. (DOCX 41 kb) [file 40409_2018_150_MOESM3_ESM.docx]
